# The role of bacterial genital infections in spontaneous preterm delivery: a case-control study

**DOI:** 10.3389/fcimb.2024.1348472

**Published:** 2024-06-18

**Authors:** Amjad Ahmadi, Mohammad Bagher Khadem Erfan, Daem Roshani, Safoura Derakhshan, Rashid Ramazanzadeh, Fariba Farhadifar, Behzad Mohsenpour, Sholeh Shahgheibi, Mozhdeh Zarei, Himen Salimizand, Bahram Nikkhoo

**Affiliations:** ^1^ Cellular and Molecular Research Center, Research Institute for Health Development, Kurdistan University of Medical Sciences, Sanandaj, Iran; ^2^ Student Research Committee, Hamadan University of Medical Sciences, Hamadan, Iran; ^3^ Department of Parasitology and Mycology, Faculty of Medicine, Kurdistan University of Medical Sciences, Sanandaj, Iran; ^4^ Health Metrics and Evaluation Research Center, Research Institute for Health Development, Kurdistan University of Medical Sciences, Sanandaj, Iran; ^5^ Environmental Health Research Center, Research Institute for Health Development, Kurdistan University of Medical Sciences, Sanandaj, Iran; ^6^ Department of Microbiology, Faculty of Medicine, Ardabil University of Medical Sciences, Ardabil, Iran; ^7^ Social Determinants of Health Research Center, Research Institute for Health Development, Kurdistan University of Medical Sciences, Sanandaj, Iran; ^8^ Department of Obstetrics and Gynecology, Faculty of Medicine, Kurdistan University of Medical Sciences, Sanandaj, Iran; ^9^ Zoonoses Research Center, Research Institute for Health Development, Kurdistan University of Medical Sciences, Sanandaj, Iran; ^10^ Deputy of research and technology, Kurdistan University of Medical Sciences, Sanandaj, Iran; ^11^ Department of Microbiology, Faculty of Medicine, Kurdistan University of Medical Sciences, Sanandaj, Iran; ^12^ Liver and Digestive Research Center, Research Institute for Health Development, Kurdistan University of Medical Sciences, Sanandaj, Iran

**Keywords:** genital infections, spontaneous preterm delivery, women, PCR, Iran

## Abstract

**Background:**

Spontaneous preterm delivery is defined as the beginning of the birth process before the 37th week of pregnancy. The presence of microorganisms in the fetal membranes is accompanied by an increase in the production of prostaglandin, one of the important factors associated with the prevalence of preterm birth. The invasion of microorganisms leads to the production of protease, coagulase, and elastase, which directly stimulate the onset of childbirth. We investigated the role of genital infections in women with preterm birth.

**Methods:**

The present case-control study was conducted in the west of Iran on 100 women with spontaneous preterm delivery (following 24 weeks of gestation and before 36 weeks and 6 days) as the case group and 100 women with normal delivery as controls. A questionnaire was applied to collect the data. Polymerase chain reaction and pathological examination of the placenta were performed.

**Results:**

The average age in women with normal delivery (30.92 ± 5.10) in women with spontaneous preterm delivery (30.27 ± 4.93). The prevalence of *Chlamydia trachomatis*, *Neisseria gonorrhea, Listeria monocytogenes, and Mycoplasma genitalium* infections was zero in both groups. The highest prevalence of *Gardnerella vaginalis was* 19 (19%) in the case group and *Ureaplasma parvum* 15 (15%) in the control group. Also, Placental inflammation was zero in controls and 7(7%) in the patient group. There was a significant relationship between *Gardnerella vaginalis* bacteria and spontaneous preterm delivery.

**Conclusion:**

The results of our study showed that except for *Gardnerella vaginalis* bacteria, there is no significant relationship between the above bacterial infections and spontaneous preterm birth. Moreover, despite the significant reduction in the prevalence of many sexually transmitted infections in this research, it is still suggested to increase the awareness of people, including pregnant women, about the ways it can be transmitted by gynecologists and health and treatment centers.

## Introduction

Spontaneous preterm delivery is defined as the beginning of the birth process before the 37th week of pregnancy (Collins et al., 2023; Smith, 2023). Spontaneous preterm delivery is one of the most important issues that involves obstetricians and gynecologists because the care and treatment of the complications of the birth of a preterm baby incur a huge cost every year and sometimes cause irreparable emotional and psychological damage to families. Today, a high percentage of pregnant women have preterm birth (4.9%–6.2%), which has caused a lot of problems and costs for the health system (Hoffman et al., 2016; Behboudi-Gandevani et al., 2023). Complications caused by spontaneous preterm delivery in babies include necrotizing enterocolitis, hyaline membrane disease, brain’s intraventricular hemorrhage, and if the baby survives, deafness, vision disorders, seizures, and neurological disorders (Stamm et al., 2005). In general, the prevalence of preterm birth is about 10%, and despite extensive studies and scientific advances, this phenomenon is still a big challenge for experts (Vakilian et al., 2015; Muchie et al., 2020; Reddy et al., 2022).In the past few years, uterine infections have been raised as one of the causes of preterm delivery, and it is estimated that more than two-thirds of preterm births are due to intrauterine infection (Haywood et al., 1994). Evidence suggests that infections and chronic inflammation are the main causes of preterm birth (McDonald and Yarnell, 2003; Megli and Coyne, 2022). The presence of microorganisms in the fetal membranes is accompanied by the increased production of prostaglandin, which is one of the important factors associated with the prevalence of preterm delivery. The invasion of microorganisms results in the generation of protease, coagulase, and elastase, which directly stimulate the onset of childbirth (McGregor et al., 1986; Daskalakis et al., 2023). In some studies, such as the study by Seong Jin Choi et al., no relationship was observed between genital infections and preterm birth (Choi et al., 2012). In a review conducted by Nadeau et al. on the role of infection in preterm labor, bacterial and viral infections were not correlated with preterm labor (Nadeau et al., 2016). In an investigation, unlike the two previous studies conducted by Chung et al., genital mycoplasmas had a significant relationship with preterm birth (Chung et al., 2012). According to the contradictory studies regarding the role of genital infections with preterm delivery, we investigated the role of these infections in women with spontaneous preterm delivery.

## Materials and methods

The present case-control study was conducted in the west of Iran on 100 women with spontaneous preterm delivery (following 24 weeks of gestation and before 36 weeks and 6 days) as the case group and 100 women with normal delivery as controls. The age range of the subjects was 18–40 years and were referred to Besat Hospital of Sanandaj, Iran, during 2018–2023. All the patients signed a written consent form and the project was explained to them in detail. The inclusion criteria included not taking antibiotics and being sexually active. The exclusion criteria were: multiple pregnancies, high blood pressure, diabetes and immunodeficiency, fetal abnormalities, placenta Previa, placenta accrete spectrum, vasa Previa, prior fundal hysterectomy, preterm rupture of membranes, oligohydramnios or polyhydramnios, preeclampsia with severe features, and intrahepatic cholestasis of pregnancy with high total bile acid levels. A questionnaire was applied to collect the data. Polymerase chain reaction (PCR) was used on cervical swab samples and the placenta was examined pathologically. For PCR, first, an endocervical swab was taken from all women. Next, specimens were placed in sterilized phosphate-buffered saline and stored at -20°C until DNA extraction. In addition, the complete placenta specimens were transported to the pathology laboratory in containers containing 10% formalin for pathology examination.

### DNA extraction

DNA extraction was performed according to the guidelines of the ROCH kit. After DNA extraction, the samples in 1.5 ml microtubes were kept at the temperature of -20 C° until PCR.

### PCR for detecting genital infections

The specific primers for the 16S ribosomal genes of the bacterial genome are presented in **Table 1**. The total volume of the PCR reaction was 25 microliters using the pre-made PCR master mix (CinnaGen, Iran). Separation of PCR products was done by electrophoresis in 1.5% gel agarose, visualized by UV light, and photographed.

**Table 1:** PCR primers and cycling parameters used to detect sexually transmitted bacteria.

Bacterium	Primer/sequences (5 - 3)	PCR condition	PCR product (bp)	Reference
*Ureaplasma* *urealyticum*	F: TTTGCAAAACTATAAATAGAC AC R: TTTGTTGTTGCGTTTTCTG	30 sec 94°C, 30 sec 60°C, 1 min 72°C	363	(Zeighami et al., 2009)
*Ureaplasma* *parvum*	F:AATAAATCTTAGTGTTCATATTTTTTTA R: GTAAGTGCAGCATTAAATTCAATG	1 min 94°C, 1 min 52°C, 1 min 72°C	327	(Zeighami et al., 2009)
*Mycoplasma* *hominis*	F: CAATGGCTAATGCC GATACG C R: GGTACCGTCAGTCTGCAAT	35 sec 95°C, 35 sec 55°C, 30 sec 72°C	334	(Maleki et al., 2013)
*Mycoplasma* *genitalium*	F: AGTTGATGAAACCTTAACCCC TTG G R: CCGTTGAGGGGTTTTCCATTT TTGC	60 sec 94°C, 30 sec 64°C, 60 sec 72°C	282	(Ramazanzadeh et al., 2016)
*Chlamydia trachomatis*	F: TGGCGGCGTGGATGAGGCAT R:CTCAGTCCCAGTGTTGGCGG	30 sec 94°C, 30 sec 64°C, 25 sec 72°C	300	(Ahmadi et al., 2020)
*Neisseria gonorrhoeae*	F: GCA TACGTCTTGAGAGRGAA R: ACACTCGAGTCACCCAGTTC	30 sec 94°C, 30 sec 64°C, 25 sec 72°C	482	(Ahmadi et al., 2022a)
*Gardnerella vaginalis*	F: TTTCACCAGCTGTATTAGAAGTA R: GTTCCCTGAACATTATCTTTGAT	60 sec 94°C, 60 sec 55°C, 30sec 72°C	330	(De Backer et al., 2007)
*Streptococcus agalactiae*	F: TTTCACCAGCTGTATTAGAAGTA R: GTTCCCTGAACATTATCTTTGAT	60 sec 94°C, 60 sec 55°C, 60 sec 72°C	153	(Ahmadi et al., 2018)
*Listeria monosytogens*	F: GCTGAAGAGATTGCGAAAGAAG R:CAAAGAAACCTTGGATTTGCGG	30 sec 94°C, 30 sec 58°C, 45 sec 72°C	370	(Ahmadi et al., 2022b)

### Pathologic examination

Seven tissue sections (Includes 2 sections of umbilical cord and 5 sections of placenta and curtain) were separated from samples of both groups and were kept in 10% formalin and placed inside the tissue processor for 16 hours. Afterward, the samples were removed from the device and blocked by paraffin. The tissue blocks were kept in the freezer for 1–2 hours before cutting. Next, a 3-micrometer section was prepared from the samples and placed on a slide, and to fix them and remove the paraffin, they were placed in a 70°C incubator. Finally, slides were stained with the hematoxylin-eosin method.

### Statistical analysis

Data were entered in the SPSS software and were summarized as tables and diagrams by mentioning percentages. The chi-square test and t-test were applied for comparing different qualitative variables and means between the two groups, respectively. In all these steps, a level of 5% was considered significant. SPSS 22 was employed for statistical analyses.

## Results

The average age in women with normal delivery and spontaneous preterm delivery was 30.92 ± 5.10 and 30.27 ± 4.93 years, respectively. The mean pregnancy termination age was 38.76 ± 0.58 weeks in women who had normal delivery and 33.27 ± 2.33 weeks in women with spontaneous preterm delivery. Alcohol consumption was zero for all groups. The highest education level in all groups was a high school degree. The most prevent pregnancy method in all groups was the withdrawal method. The prevalence of *Chlamydia trachomatis*, *Neisseria gonorrhea*, *Listeria monocytogenes*, and *Mycoplasma genitalium* infections was zero in both groups. A summary of the results is presented in **Table 2**.

**Table 2:** Demographic data, risk factors, and genital infections in women with spontaneous preterm delivery and women with normal delivery.

Parameter		Normal delivery n=100	Spontaneous preterm delivery n=100	P-value
Age		30.92 ± 5.10	30.27 ± 4.93	0.33
pregnancy termination age		38.76 ± 0.58	33.27 ± 2.33	0.00
Education	Illiterate	3 (3%)	9 (9%)	0.36
	Elementary school	26 (26%)	25 (25%)	
	Junior high school	16 (16%)	20 (20%)	
	High school	32 (32%)	28 (28%)	
	University education	23 (23%)	18 (18%)	
Habitat	City	74 (74%)	68 (68%)	0.35
	Village	26 (26%)	32 (32%)	
Job	Employee	10 (10%)	11 (11%)	0.81
	Housekeeper	90 (90%)	89 (89%)	
Smoking		0 (0%)	0 (0%)	–
Alcohol		0 (0%)	0 (0%)	–
Smoking in spouse		26 (26%)	18 (18%)	0.17
History of genital infection during pregnancy		13 (13%)	26 (26%)	0.02
History of genital infection before pregnancy		23 (23%)	19 (19%)	0.48
History of urinary tract infection (UTI)		11 (11%)	9 (9%)	0.63
History of spontaneous preterm birth		3 (3%)	6 (6%)	0.30
Prevention methods	Withdrawal	65 (65%)	76 (76%)	0.01
	Oral Contraceptive Pills (OCP)	12 (12%)	13 (13%)	
	Intrauterine device (IUD)	14 (14%)	2 (2%)	
	Barrier	9 (11.9%)	9 (5.5%)	
*Chlamydia trachomatis (C. trachomatis)*		0 (0%)	0 (0%)	–
*Neisseria gonorrhea (N. gonorrhea)*		0 (0%)	0 (0%)	–
*Listeria monocytogenes (l. monocytogenes)*		0 (0%)	0 (0%)	–
*Mycoplasma genitalium (M. genitalium)*		0 (0%)	0 (0%)	–
*Mycoplasma hominis (M. hominis)*		4 (4%)	3 (3%)	0.70
*Ureaplasma parvum (U. parvum)*		15 (15%)	14 (14%)	0.84
*Ureaplasma urealeticum (U. urealeticum)*		3 (3%)	0 (0%)	0.08
*Gardnerella vaginalis (G. vaginalis)*		9 (9%)	19 (1%)	0.04
*Streptococcus agalactiae (S. agalactiae)*		5 (5%)	3 (3%)	0.47

The results of bacterial co-infection in two groups showed (**Table 3**) that the highest frequency was related to *U. parvum* & *G. vaginalis* co-infection in both groups.

**Table 3:** Frequency of the co-existence of evaluated bacteria.

Bacteria	Normal delivery n=100	Preterm delivery n=100
*M. hominis & U. parvum*	3 (3%)	0 (0%)
*M.hominis & G. vaginalis*	1 (1%)	0 (0%)
*M.hominis & S. agalactiae*	1 (1%)	0 (0%)
*U. urealeticum & G. vaginalis*	1 (1%)	0 (0%)
*U. parvum & G. vaginalis*	5 (5%)	2 (2%)
*U. parvum & S. agalactiae*	1 (1%)	0 (0%)
*S. agalactiae & G. vaginalis*	0 (0%)	1 (1%)
*U. parvum & S. agalactiae & M. hominis*	1 (1%)	0 (0%)

The analysis of all genital infections in this study showed that 35 (35%) people in the group of women with normal delivery and 26 (26%) people in the group of women with spontaneous preterm delivery had infections. Also, there was the highest frequency of co-infection in the group of women with spontaneous preterm delivery **Table 4**.

**Table 4:** **​** Total frequency of genital infections in female subjects with normal delivery and women with spontaneous preterm delivery.

Groups	Negative infection	Positive infection	A type of infection	Two types of infection	Three types of infection
Normal delivery	65 (65%)	35 (35%)	25 (25%)	9 (9%)	1 (1%)
Spontaneous preterm delivery	74 (74%)	26 (26%)	23 (23%)	3 (3%)	0 (0%)

**Figure 1:**
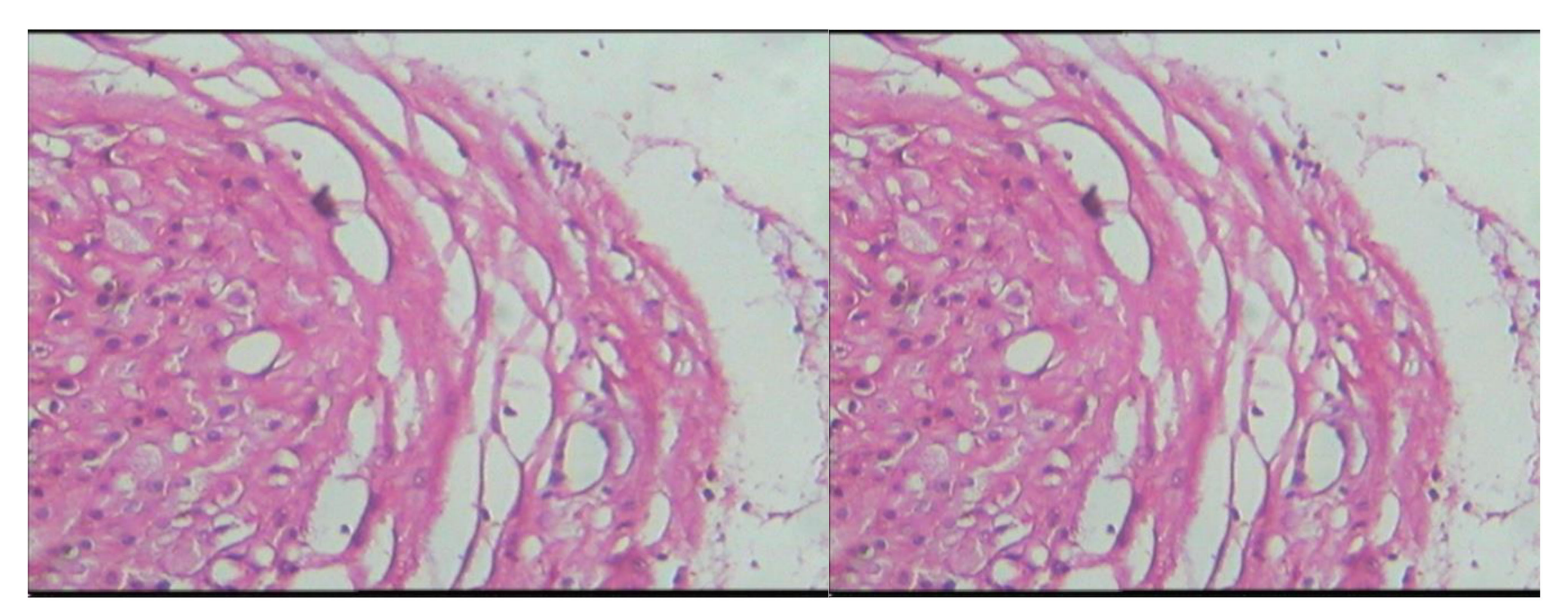
Microscopic slide image of the amniotic membrane of placenta specimens from women who had spontaneous preterm delivery.

**Figure 2:**
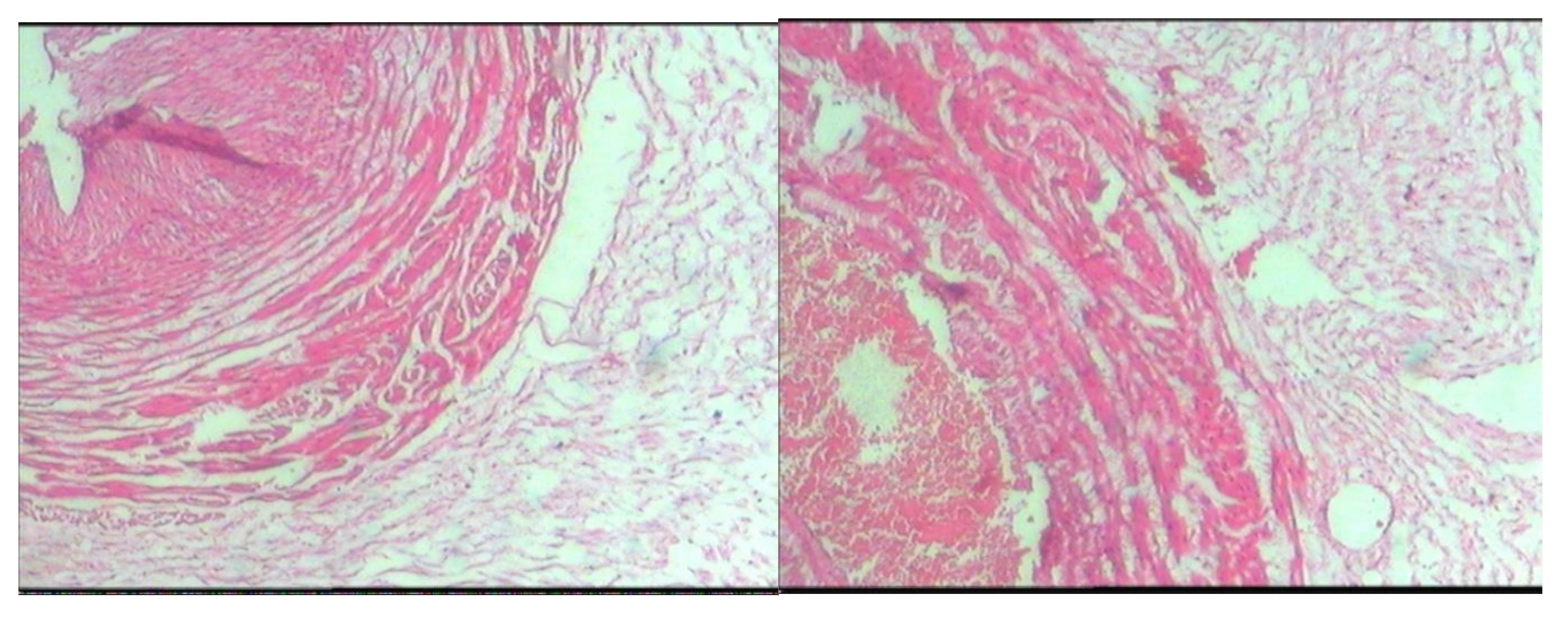
Microscopic slide image of the amniotic membrane of placenta specimens from women who had normal delivery.

Examining the pathology results of the placenta samples of the patients in both groups showed that there was no inflammation in the placenta samples in controls, but in the patient group, seven cases had symptoms of inflammation, so their analysis showed a significant relationship between placenta inflammation and Preterm delivery, but this inflammation has nothing to do with infections isolated from vaginal samples in this study and it was probably due to other infections such as viruses and protozoa (**Figures 1**, **2** and **Table 5**).

**Table 5:** Frequency of the inflammation of placenta specimens in women who had normal delivery and women who had spontaneous preterm delivery.

Variable		Spontaneous preterm delivery	Normal delivery	P-value
Placental inflammation	The presence of inflammation	7 (0%)	0 (0%)	0.007
	Absence of inflammation	93 (93%)	100 (100%)	

The matching of the pathology results of the placenta samples with the molecular results of the genital swabs in the groups showed that in only one of the samples, the patients with *U. parvum* infection were also positive for placental inflammation, and in other samples, the presence of genital infections did not match with placental inflammation. Therefore, there was no direct relationship between genital infections and placenta inflammation (**Table 6**).

**Table 6:** Co-frequency of bacteria in the vaginal swab samples of women with spontaneous preterm delivery and inflamed placentas.

Bacteria	Spontaneous preterm delivery (n:100)	Inflammation of the placenta (n:7)	P-value
*C. trachomatis*	0 (0%)	0 (0%)	–
*M. hominis*	3 (3%)	0 (0%)	0.81
*M. genitalium*	0 (0%)	0 (0%)	–
*U. parvum*	14 (14%)	1 (1%)	0.66
*U. urealeticum*	0 (0%)	0 (0%)	–
*N. gonorrhea*	0 (0%)	0 (0%)	–
*S. agalactiae*	3 (3%)	0 (0%)	0.81
*G. vaginalis*	9 (9%)	0 (0%)	0.53
*L. monocytogenes*	0 (0%)	0 (0%)	–

## Discussion

Spontaneous preterm delivery is one of the most important issues that involve obstetricians and gynecologists because the care and treatment of the complications of preterm birth incur a huge cost every year and sometimes cause irreparable emotional and psychological damage to families. Today, a high percentage of pregnant women experience preterm birth before reaching the end of pregnancy (Cobo et al., 2020; Collins et al., 2023). Despite the use of birth control pills, the prevalence of spontaneous preterm delivery has not decreased significantly (Danforth, 2008). Different factors are involved in spontaneous preterm delivery, including multiple births, diabetes mellitus, cardiac diseases, urinary tract infections (UTIs), renal diseases, and recurrent pregnancy loss, of which UTIs are responsible for 25%-40% of all cases (Pangastuti et al., 2019; Baer et al., 2021). Infections highly affect human reproductive health, and infections during pregnancy lead to stillbirth or prematurity or can be vertically transmitted to the fetus and cause severe disease and congenital infection (Megli and Coyne, 2022). Chronic inflammation and infection are some important causes of spontaneous preterm delivery (Coussons-Read et al., 2012; Barinov et al., 2022). Furthermore, intrauterine and genital tract infections, leading to rupture of membranes, can cause spontaneous preterm delivery (Cunnington et al., 2013; Tedesco et al., 2020; Megli and Coyne, 2022). In 2012, Seong et al. performed PCR to detect genital infections in preterm labor in a case-control study on 126 women with preterm labor and 91 women who had natural labor. They used vaginal swab samples to perform the test. Their results revealed that 17.6%, 0%, 11.5%, 0%, 0%, and 0% cases in the control group and 3.8%, 3.8%, 16.7%, 0%, 0%, and 0% in the patient group were infected with *S. agalactiae, M. hominis, M. genitalium, U. urealeticum, N. gonorrhea*, and *Trichomonas vaginalis*, respectively. Therefore, they did not find a relationship between the mentioned infections and preterm birth (Choi et al., 2012). The results of Seong et al. were similar to our research and they found no significant association between genital infections and preterm delivery. However, we observed a significant relationship between *G. vaginalis* and delivery. In 2014, Kwak et al. conducted a cross-sectional study on 184 women to assess the relationship of preterm birth with genital infection with *U. urealeticum* and *M. hominis*. They took a cervical swab sample from each person and observed that 62% of the women were infected with *U. urealeticum* and 7% of women were infected with *M. hominis.* They concluded that there is a relationship between *U. urealeticum* and preterm delivery (Chung et al., 2012). The results of their research were contrary to our findings, which might result from the type of study and the method of conducting tests in this research because they used culture, while we applied a more sensitive and accurate molecular method to detect the infection. In 2008, Robert et al. performed a blood culture test on 351 umbilical cord blood specimens to determine the role of genital infection with *U. urealeticum* and *M. hominis* in spontaneous preterm birth. They observed that 23% of blood cultures were positive for *U. urealeticum* and *M. hominis.* These researchers concluded that there is a significant relationship between *U. urealeticum* and *M. hominis* with spontaneous preterm birth (Goldenberg et al., 2008). The results of this research were similar to the findings of Kwak et al. and were in contrast to our study. This discrepancy may result from the use of culture instead of the molecular method, just like the results of Kwak et al. In 2020, Ahmadi et al. conducted PCR to detect genital *C. trachomatis* infection in women who had spontaneous preterm delivery, a case-control study on 75 women who had spontaneous preterm delivery and 75 women who had normal delivery. They used vaginal swab samples to perform the test and after conducting the test, they observed that 7 (9.33%) of the controls and 2 (2.67%) of the patient group participants were infected with *C. trachomatis*. Therefore, they did not find a relationship between the mentioned infections and spontaneous preterm delivery (Ahmadi et al., 2020). Although the results of this study were similar to our research, the noteworthy point was the significant reduction of chlamydia infection and its zero prevalence in our study. Overall, the results of our investigation showed no significant relationship between the above bacterial infections and spontaneous preterm birth. Although in this study, the prevalence of *G. vaginalis* bacteria was higher in women who had spontaneous preterm delivery than in women who had normal delivery, also the results of examining the pairs of patients showed that the studied bacterial infections did not directly play a role in spontaneous preterm birth, but the inflammatory reactions of the immune system against them may be one of the factors that cause spontaneous preterm birth, and it is better to understand the role of inflammatory cytokines in These infections should be considered and investigated at the same time. However, the decrease in its frequency in inflamed placentas diminishes its role in preterm delivery. However, it seems that due to the high prevalence of genital infection in the patient group (26%) compared to the control group (13%) in Table No. 2, the role of other infections in preterm labor such as viruses and protozoa should be given more attention by gynecologists. However, more detailed studies such as the simultaneous examination of vaginal and amniotic fluid samples are necessary for the presence of bacterial infections. Also, although there significant relationship was detected between the prevalence of urinary tract infection and preterm delivery, from a clinical point of view, its increase is very significant in both groups, and it seems that in order to reduce these infections, preventive and conscious measures should be taken among pregnant women.

## Conclusion

The results of our study showed that except for *G. vaginalis* bacteria, there is no significant relationship between the above bacterial infections and spontaneous preterm birth. However, the cause-and-effect relationship for most bacterial infections and its relationship with spontaneous preterm delivery is still controversial. Also, despite the significant reduction in the prevalence of many sexually transmitted infections in this research, it is suggested to increase the awareness of pregnant women about the ways it can be transmitted by gynecologists and health and treatment centers. It is also better to consider the diagnosis of other infections such as viruses, fungi, and protozoa.

## Data availability statement

The raw data supporting the conclusions of this article will be made available by the authors, without undue reservation.

## Author contributions

AA: Supervision, Writing – review & editing. MK: Writing – review & editing. DR: Writing – review & editing, Formal analysis. SD: Methodology, Writing – review & editing. RR: Investigation, Writing – review & editing. FF: Data curation, Writing – review & editing. BM: Writing – review & editing, Data curation. SS: Resources, Writing – review & editing. MZ: Resources, Writing – review & editing. HS: Resources, Writing – review & editing. BN: Writing – original draft.
